# The role of self-compassion in the relationship between resilience and negative affect

**DOI:** 10.1038/s41598-026-42585-x

**Published:** 2026-03-04

**Authors:** Timo J. Lajunen, Marios Adonis, Maria Giagkou, Mark J. M. Sullman

**Affiliations:** 1 Present Address: Department of Psychology, Norwegian University of Science and Technology, NTNU, Torgarden, Trondheim, Postboks 8900, NO-7491 Norway; 2 Department of Psychology, University of Helsinki, Helsinki, Finland; 3 School of Humanities and Social Sciences, Department of Social Sciences, University of Nicosia, Nicosia, Cyprus

**Keywords:** Negative affect, Anxiety, Stress, Depression, Resilience, Self-compassion, Health care, Psychology, Psychology

## Abstract

Third-wave psychotherapeutic approaches, which emphasise acceptance and mindfulness, have shown effectiveness in alleviating negative affect. This study examined the role of self-compassion in the relationship between resilience and negative affect, including symptoms of stress, anxiety, and depression. A cross-sectional design was employed with 494 adults from the general population. Participants completed the Depression Anxiety Stress Scale-21 (DASS-21), the Self-Compassion Scale (SCS), and the Brief Resilience Scale (BRS). Regression analyses indicated that higher resilience was associated with lower levels of negative affect and self-compassion was significantly correlated with both resilience and negative affect. Mediation analyses revealed a significant indirect effect, suggesting that self-compassion is associated with a portion of the variance in the relationship between resilience and negative affect. These findings highlight a notable association between self-compassion, resilience, and emotional distress, suggesting that self-compassion may be a relevant construct for interventions aimed at enhancing psychological wellbeing.

## Introduction

Stress, anxiety, and depression are among the most prevalent mental health disorders, affecting individuals across various age groups and demographics across the globe^1–3^. These conditions often co-occur and exhibit high rates of comorbidity, which exacerbates their severity and complicates treatment efforts. For instance, those with anxiety disorders are at a significantly increased risk of developing depression, reinforcing the critical importance of addressing comorbidity in mental health^2,4,5^. According to the Diagnostic and Statistical Manual of Mental Disorders, 5th edition (DSM-5), mental health disorders exist on a spectrum, ranging from mild to severe, and they significantly impair individuals’ quality of life and daily functioning^2,6^. This highlights the urgent need for therapeutic approaches that address both the emotional and functional challenges posed by these disorders.

In response to this need, Cognitive Behavioural Therapy (CBT) and third-wave psychotherapies (e.g., Mindfulness-Based Cognitive Therapy, Compassion-Focused Therapy, and Acceptance and Commitment Therapy), which emphasise mindfulness, resilience, and self-compassion, have gained recognition for their efficacy in alleviating symptoms of anxiety, stress, and depression^7,8^. The aim of these treatments is to build a more adaptable connection with one’s thoughts and emotions, enhancing psychological flexibility and emotional regulation^9–11^. Within this framework, self-compassion and resilience have emerged as key factors in emotional regulation, enhancing individuals’ ability to adapt to adversity and reducing psychological distress^12–15^.

### Self-compassion

Self-compassion, originating from Buddhist philosophy, refers to the practice of extending kindness, care, and non-judgemental understanding toward oneself, particularly during periods of failure or adversity^13,14^. This contrasts with the common tendency to respond to personal shortcomings with harsh self-criticism and judgement. According to Neff^16^, self-compassion is comprised of three core components: mindfulness (rather than over-identification), recognition of shared human experience (as opposed to isolation), and self-kindness (as opposed to self-judgement). Together, these elements foster emotional balance and promote healthier emotional responses^12^.

Self-compassion fosters psychological resilience by promoting adaptive emotion regulation strategies, such as mindfulness and self-kindness, while mitigating maladaptive strategies like self-judgement and rumination^17,18^. This shift in emotional regulation is crucial, as individuals who engage in self-compassion tend to report lower levels of negative mental health symptoms, including depression and anxiety^19,20^. A meta-analysis has identified a robust negative relationship between self-compassion and various mood disorders, indicating that greater self-compassion is consistently linked with reduced anxiety and depression across populations^21^. Self-compassion has been found to be related to more favourable mental health outcomes, such as lower levels of anxiety, stress, and depression^22,23^. Furthermore, self-compassion has been recognised as a protective factor in high-stress environments, such as among combat veterans, where it helps buffer against the psychological impacts of morally injurious experiences^19^.

Interventions designed to cultivate self-compassion have been linked to significant enhancements in mental health, resulting in large decreases in psychological distress. This indicates that self-compassion may be a crucial protective factor in managing negative emotional states^12^. For example, mindfulness-based programs that enhance self-compassion have shown promising results in alleviating stress and enhancing overall well-being^24^.

### Resilience

Resilience, characterised as the ability to adapt to and surmount adversity, stress, or trauma, is increasingly recognised as a critical factor in maintaining psychological well-being, particularly in the face of adversity^25,26^. Resilient individuals exhibit greater emotional strength and flexibility when facing life’s challenges, enabling them to maintain psychological well-being despite adversity^27,28^. Traditionally, resilience was viewed as an inherent quality that individuals possessed, often linked to stable personality traits. However, contemporary studies emphasise that resilience is not merely an innate characteristic but rather a complex interplay of various factors that can be enhanced through targeted interventions and supportive environments^29,30^.

Higher resilience is correlated with a reduced risk of developing depression and anxiety, as well as improved coping mechanisms^31–33^. Moreover, resilience has been linked to enhanced emotional regulation strategies, including self-compassion and mindfulness, which further contribute to mental well-being^8^. However, the precise function of self-compassion in the resilience-negative affect relationship remains insufficiently understood, warranting further investigation.

### Negative affect

For the purposes of this investigation, negative affect was operationalised as the symptomatic distress captured by the DASS-21. This instrument was selected over more general mood measures (e.g., the PANAS) due to its clinical focus on the core symptoms of depression, anxiety, and stress, which aligns with the study’s primary aim of investigating pathways to psychological distress. The Tripartite Model of Anxiety and Depression^34^ was chosen as the guiding theoretical framework because it provides a direct and empirically supported conceptualisation of the shared and unique components of these states, positing a common underlying factor of negative affect that the DASS-21 total score effectively represents.

Negative affect refers to a range of negative emotional states, including anxiety, depression, and stress, which often co-occur and contribute to emotional distress^35,36^. The Tripartite Model serves as a theoretical foundation for understanding the nuances within these states. According to this model, depression and anxiety share a common underlying component of general negative affect while also possessing unique characteristics. Specifically, depression is distinguished by anhedonia (i.e., low positive affect), whereas anxiety is distinguished by physiological hyperarousal. Stress, within this context, manifests as a combination of irritability, persistent tension, and a low threshold for becoming upset. Research has established that heightened negative affect significantly impairs mental health, particularly in stressful situations, by exacerbating feelings of distress and impeding flourishing, making it a critical component in mental health research^37–41^.

Within this framework, the current research focuses specifically on symptoms of stress, anxiety, and depression, widely recognised as key indicators of poor mental health and impaired functioning^2,42^. Through the examination of these symptoms, we seek to contribute to the expanding body of research concerning negative affect and its adverse impacts on mental health.

### Theoretical framework for the proposed mediation

Although both self-compassion and resilience are established protective factors against negative affect, the nature of their relationship requires theoretical clarification. The present study is grounded in contemporary, process-oriented views of resilience^29^. Within this framework, resilience is conceptualised as a relatively distal psychological resource that enables individuals to mobilise more proximal, adaptive regulatory processes when facing adversity^8,31^. We posit that self-compassion represents one such key proximal process. Specifically, we propose that individuals with higher resilience are better equipped to respond to stressors and negative emotions with greater self-kindness, mindful awareness, and a sense of common humanity, rather than with self-criticism and isolation^12^. In turn, these self-compassionate responses are theorised to more directly attenuate symptoms of depression, anxiety, and stress. This theoretical perspective justifies specifying a mediation model in which resilience, as an upstream protective factor, exerts its influence on negative affect indirectly through the pathway of enhanced self-compassion.

### The current study

Although the independent, salutary effects of resilience^43,44^ and self-compassion on mental health are well-documented, with self-compassion consistently associated with lower levels of stress, anxiety, and depression^45^, the precise nature of their interplay remains insufficiently specified. Prior research has confirmed a positive association between the two constructs and has begun to explore their combined influence. For instance, some studies have positioned resilience as a mediator in the pathway from self-compassion to reduced depressive symptoms^8^. However, this formulation treats resilience as a consequence of self-compassion. The reverse mediational pathway has not yet been formally tested. This alternative model conceptualises self-compassion (understood as an adaptive stance toward the self in times of suffering) as the mechanism through which an individual’s resilience exerts its protective effects.

Clarifying this directional pathway is of considerable theoretical and clinical importance. Theoretically, it would advance a process-oriented model of resilience, reframing it from a static trait to a dynamic capacity that activates proximal regulatory skills to be effective. Clinically, identifying self-compassion as a key mediator would provide a specific, actionable target for interventions. Therapeutic strategies could then focus on cultivating self-compassion as a primary mechanism for leveraging an individual’s existing resilient capacities to improve mental health outcomes. Grounded in process-oriented views of resilience as a distal resource that facilitates proximal adaptive processes, we specify a model in which resilience mitigates psychological distress indirectly through the pathway of enhanced self-compassion.

This study was designed to address this specific gap by testing the specified mediational model. Specifically, we hypothesise that:

1. Resilience will be negatively associated with negative affect and positively associated with self-compassion.
2. Self-compassion will be significantly associated with both resilience and negative affect.
3. Self-compassion will statistically mediate the association between resilience and negative affect.

This study seeks to enhance our understanding of the interrelationship between resilience and self-compassion as factors associated with psychological distress. Additionally, by testing the proposed mediational model, this study aims to replicate prior findings regarding the associations between these variables while offering novel insights into the potential mechanisms through which resilience operates.

## Method

This study employed a cross-sectional survey design.

### Participants and procedure

A non-probability snowball sampling method was used for recruitment. Sixty university student volunteers were each provided with ten questionnaire packets to distribute within their social networks. Volunteers were instructed to recruit individuals from various age groups, aiming for two participants per age bracket (18–25, 26–35, 36–45, 46–55, and 56+). Inclusion criteria for the study were age 18 and above, fluency in the Greek language, and the ability to provide informed consent. There were no other exclusion criteria for the study. Each packet contained measures of depression, anxiety, stress, self-compassion, and resilience, along with a consent form, detailed instructions, and a sealed return envelope. Participants provided written informed consent before completing the questionnaire and returning it in the sealed envelope provided. The study was approved by the University of Nicosia Research Ethics Committee and was conducted in accordance with the ethical standards of the Declaration of Helsinki.

### Participants

Of the 600 questionnaire packets distributed, 494 (82%) were returned. The data were then screened for adherence to inclusion criteria, completeness, duplication, and response quality. No duplicate submissions were identified. A visual inspection was conducted to screen for obvious irregularities, such as patterned responding (e.g., straight-lining), multiple responses per item, or missing pages. It must be noted that no formal indices of careless or inattentive responding (e.g., infrequency items) were embedded in the survey, which precluded a more systematic screening. Following this process, six packets were excluded due to substantial incompleteness (defined as > 10% of items missing), resulting in a final analytical sample of 488 participants.

The final sample (*N* = 488) included 299 women (61.3%), 189 men (38.7%), with ages ranging from 18 to 81 years (M = 34.7). Regarding education, 26% of participants had completed primary or secondary education, almost half had a university degree (48.6%), and 25% had pursued postgraduate studies. One participant reported having no formal education. Almost half of participants indicated they were single (51%), 39% were married, and 9.6% were separated, divorced, or widowed. Socioeconomic status was reported using a 10-point Likert scale, where 1 represented the lowest status and 10 the highest. The distribution of socioeconomic status was normally distributed (M = 5.94, SD = 1.36).

### Measures

#### Depression anxiety stress scale 21

The Depression Anxiety Stress Scale-21 (DASS-21)^42^ is a widely used self-report questionnaire that measures three related negative emotional states: stress, anxiety, and depression. This 21-item scale comprises three 7-item subscales, each assessing one of the emotional states. Participants reported the degree to which they have experienced each state during the last week on a 4-point Likert scale. In addition to the three DASS-21 subscale scores (depression, anxiety, stress), we computed a composite negative affect score by summing the three subscale sum scores (range: 0–126), such that higher scores indicated higher overall negative affect. This operationalisation follows common practice of using the DASS total score as an index of general negative emotionality or distress that reflects the shared variance among the three subscales.

This study utilised the validated Greek version of the DASS-21^46^. Cronbach’s alpha demonstrated excellent reliability for the overall scale (α = 0.95) and each subscale: Stress (α = 0.90), Anxiety (α = 0.88), and Depression (α = 0.89).

#### Self-compassion scale (SCS)

The Self-Compassion Scale (SCS) is a 26-item instrument that measures an individual’s general propensity to exhibit self-compassion in the face of personal inadequacies, external challenges, or failures^13^. The SCS directly assesses the frequency with which individuals engage in thoughts, emotions, and behaviours reflecting different facets of self-compassion. The scale comprises six subscales, three measuring positive aspects of self-compassion and three measuring negative (or less self-compassionate) tendencies. Higher scores on the SCS indicate a greater tendency to be compassionate and understanding toward oneself during difficult times. For the primary analyses, the total SCS score was used. This approach aligns with the theoretical conceptualisation of self-compassion as a single, superordinate construct reflecting the integrated balance of its positive and negative components^16^. While the distinct roles of the subscales represent an important area of inquiry, the total score provides the most direct test of our hypothesis concerning the overall capacity for self-compassion as a mediator. In the current study, the SCS demonstrated strong internal consistency (α = 0.84).

#### Brief resilience scale (BRS)

The Brief Resilience Scale (BRS) is a concise, six-item measure of resilience^47^. The BRS consists of three positively worded and three negatively worded items, which are reverse-coded before calculating a total score. Respondents express their degree of agreement with each statement, reflecting their resilient attitudes and behaviours, on a Likert scale^48^. Higher BRS scores indicate a greater capacity to bounce back from stressful experiences and adversity. In the current research, the BRS exhibited strong internal consistency (α = 0.81).

### Data analysis

An a priori power analysis was conducted to determine the required sample size for the primary mediation analyses, targeting 80% power at an α level of .05. A Monte Carlo simulation for a simple mediation model was performed using the method of Schoemann et al.^49^. Based on effect sizes from comparable literature, we specified moderate standardised path coefficients (a = .30, b = − .30) and a small direct effect (c’ = 0.10). The simulation indicated that a sample of approximately *N* = 113 would be required to detect the indirect effect with 80% power. Our final analytical sample of *N* = 488 therefore provided excellent statistical power (> 0.99) for the primary mediation analyses. Power was also well in excess of requirements for the secondary regression and bivariate analyses.

All statistical analyses were conducted on the final sample of 488 participants using a two-tailed α level of 0.05. Descriptive statistics were computed for all study variables, and independent-samples t-tests were used to examine sex differences in depression, anxiety, stress, self-compassion, and resilience. Pearson correlations were then calculated to assess the associations among the primary variables; for these bivariate analyses, item-level missing values were handled using pairwise deletion. To test the study’s hypotheses regarding the associations between resilience, self-compassion, and negative affect, a series of hierarchical linear regressions were conducted. For all regression and subsequent mediation analyses, cases with any missing data on the variables included in the model were excluded listwise.

To examine the mediating role of self-compassion (Hypothesis 3), four mediation models were estimated using the PROCESS 3.3 macro for SPSS^50^. Models tested resilience as the predictor and self-compassion as the mediator, with the composite negative affect score and each of the three DASS-21 subscales (depression, anxiety, and stress) as the respective outcomes. All models controlled for participant age and sex. The significance of indirect effects was determined using 5,000 bootstrap samples to obtain bias-corrected 95% confidence intervals.

## Results

### Descriptive statistics and sex differences

Table 1 provides the descriptive statistics for all study variables: depression, anxiety, stress, self-compassion, and resilience. Independent samples t-tests were conducted to explore potential sex differences in these variables. As shown in Table 2, women reported significantly higher levels of stress, anxiety, and depression compared to men. Conversely, men scored significantly higher on self-compassion. There were no significant sex differences observed for resilience.

**Table 1:** Descriptive statistics.

Measure	N	M	SD	Range
Depression	471	4.56	4.62	0–21
Anxiety	482	3.69	4.47	0–21
Stress	482	6.67	5.03	0–21
Self-compassion	466	82.29	14.93	35–127
Resilience	490	14.74	3.31	4–20

Independent samples t-tests revealed significant gender differences on several variables (see Table 2). Specifically, women reported significantly higher levels of depression, anxiety, and stress, while men reported significantly higher levels of self-compassion. No significant gender difference was found for resilience.

**Table 2:** T-tests for Sex Differences.

	Sex	N	Mean	SD	*t*	*p*	Cohen’s d
Depression	Female	285	5.09	4.97	t(449) = 3.36	*p*<.01	0.31
	Male	182	3.72	3.82			
Anxiety	Female	292	4.26	4.74	t(448) = 3.59	*p*<.01	0.39
	Male	186	2.84	3.87			
Stress	Female	291	7.09	5.23	t(476) = 2.34	*p*<.05	0.17
	Male	187	6.00	4.57			
Self-compassion	Female	283	80.36	15.42	t(461) =-3.38	*p*<.01	− 0.06
	Male	180	85.12	13.67			
Resilience	Female	298	14.53	3.21	t(484)=-1.70	*p*=.09	− 0.04
	Male	188	15.05	3.43			

### Correlation analyses

Pearson correlations were used to examine the associations between anxiety, depression, stress, self-compassion, and resilience. Table 3 presents the correlation matrix. As expected, anxiety, stress, and depression were strongly and positively correlated. Both self-compassion and resilience demonstrated significant negative correlations with stress, anxiety, and depression. Furthermore, self-compassion and resilience were positively correlated.

**Table 3:** Pearson correlations.

	1	2	3	4
1. Depression	-			
2. Anxiety	0.78**	-		
3. Stress	0.82**	0.80**	-	
4. Self-Compassion	− 0.54**	− 0.43**	− 0.53**	-
5. Resilience	− 0.22**	− 0.11*	− 0.20**	0.47**

*p<.05; **p<.01.

### Regression analyses for resilience and negative affect

To examine the first hypothesis, which posited that resilience would predict levels of negative affect, three separate hierarchical regression analyses were conducted. In each respective analysis, depression, anxiety, and stress were designated as the dependent variables. Age and sex were included as covariates in the first step, followed by the inclusion of resilience in the subsequent step.

The results supported the hypothesis. Resilience was a significant predictor of depression, F(1, 467) = 23.21, *p* < .001, explaining 5% of the variance (β = − 0.22). Similarly, resilience was a significant predictor of anxiety, F(1, 478) = 6.33, *p* < .05, explaining 1% of the variance (β = − 0.11), and stress, F(1, 478) = 20.36, *p* < .001, explaining 4% of the variance (β = − 0.20). Furthermore, resilience explained 22% of the variance (β = 0.47) in self-compassion, F(1, 464) = 129.25, *p* < .001. These findings remained significant after controlling for age and gender (Table 4).

**Table 4:** Regression analysis explaining negative affect with resilience and self-compassion.

	Variable			B	β	t	*p*
DV: Depression							
Step 1	*R*^2^= 0.02, *F*(2, 453) = 4.58, *p*< .05						
	Sex			-1.29	− 0.14	-2.91	0.00
	Age			− 0.01	− 0.03	− 0.73	0.47
Step 2	*ΔR*^2^= 0.04, *F*(3, 452) = 9.72, *p*< .001						
	Sex			-1.17	− 0.12	-2.69	0.01
	Age			− 0.01	− 0.02	− 0.46	0.65
	Resilience			− 0.28	− 0.20	-4.43	0.00
DV: Anxiety							
Step 1	*R*^*2*^= 0.03, *F*(2, 465) = 6.12, *p*< .01			-1.25	− 0.14	-3.00	0.00
	Sex			− 0.03	− 0.07	-1.62	0.11
	Age						
Step 2	*ΔR*^2^= 0.01, *F*(3, 464) = 5.76, *p*< .001						
	Sex			-1.19	− 0.13	-2.87	0.00
	Age			− 0.02	− 0.07	-1.45	0.15
	Resilience			− 0.14	− 0.10	-2.22	0.03
DV: Stress							
Step 1	*R*^*2*^= 0.01, *F*(2, 464) = 2.72, *p*= .07						
	Sex			-1.01	− 0.10	-2.12	0.04
	Age			− 0.02	− 0.04	− 0.85	0.40
Step 2	*ΔR*^2^= 0.04, *F*(3, 463) = 7.56, *p*< .001						
	Sex			− 0.87	− 0.09	-1.86	0.06
	Age			− 0.01	− 0.03	− 0.58	0.56
	Resilience			− 0.28	− 0.19	-4.13	0.00
DV: Self-compassion							
Step 1	*R*^*2*^= 0.36, *F*(2, 453) = 8.38, *p*= .001						
	Sex			4.2	0.14	2.96	0.01
	Age			0.14	0.12	2.65	0.01
Step 2	*ΔR*^2^= 0.20, *F*(3, 453) = 43.56, *p*< .001						
	Sex			2.91	0.09	2.3	0.02
	Age			0.11	0.10	2.43	0.01
	Resilience			2.03	0.45	10.89	0.00

### Regression analyses with self-compassion as a correlate of negative affect

To examine the second hypothesis, which hypothesised that self-compassion would serve as a predictor of negative affect, a series of hierarchical regression analyses were performed. In each analysis, age and sex were included as covariates in the initial step, followed by the inclusion of self-compassion in the subsequent step.

As hypothesised, self-compassion was a significant predictor of depression, F(1, 490) = 238.64, *p* < .001, explaining 30% of the variance (β = − 0.55), anxiety, F(1, 490) = 135.17, *p* < .001, explaining 19% of the variance (β = − 0.44), and stress, F(1, 490) = 216.89, *p* < .001, explaining 28% of the variance (β = − 0.53). These associations remained significant after controlling for age and gender. Specifically, self-compassion explained an additional 27% of the variance in depression, 16% in anxiety, and 26% in stress (Tables 5 and 6).

**Table 5:** Regression analysis of the association between self-compassion and negative affect.

DV	*R* ^2^	F	df	B	β	t	*p*
Depression	0.29	183.66	(1, 445)	− 0.17	− 0.54	-13.55	0.00
Anxiety	0.19	105.24	(1, 454)	− 0.13	− 0.43	-10.26	0.00
Stress	0.28	178.07	(1, 456)	− 0.18	− 0.53	-13.34	0.00

**Table 6:** Regression analysis of the association between self-compassion, resilience, and negative affect.

	Variable			B	β	t	*p*
DV: Depression							
Step 1	*R*^2^= 0.03, *F*(2, 433) = 5.69, *p*< .05						
	Sex			-1.49	− 0.16	-3.27	0.00
	Age			− 0.01	− 0.03	− 0.66	0.51
Step 2	*ΔR*^2^= 0.27, *F*(3, 432) = 60.44, *p*< .001						
	Sex			− 0.76	− 0.08	-1.94	0.05
	Age			0.01	0.03	0.78	0.43
	Self-Compassion			− 0.17	− 0.53	-12.87	0.00
DV: Anxiety							
Step 1	*R*^*2*^= 0.03, *F*(2, 443) = 7.54, *p*< .01						
	Sex			-1.41	− 0.15	-3.29	0.00
	Age			− 0.03	− 0.09	-1.82	0.07
Step 2	*ΔR*^2^= 0.16, *F*(3, 442) = 35.74, *p*< .001						
	Sex			− 0.92	− 0.10	-2.34	0.02
	Age			− 0.01	− 0.03	− 0.72	0.47
	Self-Compassion			− 0.12	− 0.41	-9.44	0.00
DV: Stress							
Step 1	*R*^*2*^= 0.02, *F*(2, 444) = 3.68, *p<* .05						
	Sex			-1.23	− 0.12	-2.52	0.01
	Age			− 0.02	− 0.04	− 0.81	0.42
Step 2	*ΔR*^2^= 0.26, *F*(3, 443) = 56.78, *p*< .001						
	Sex			− 0.52	− 0.05	-1.24	0.22
	Age			0.01	0.03	0.68	0.50
	Self-Compassion			− 0.18	− 0.52	-12.66	0.00

### Mediation analysis of self-compassion

To examine the third hypothesis, which proposed that self-compassion serves as a mediator in the relationship between resilience and negative affect, four separate mediation analyses were undertaken using PROCESS 3.3^50^. The PROCESS macro employs a bootstrapping procedure (5,000 samples) to produce bias-corrected 95% confidence intervals for indirect effects.

First, a mediation model was tested with overall negative affect (composite score of stress, anxiety, and depression) as the outcome variable. Resilience was the predictor, self-compassion was the mediator, with sex and age used as covariates. The results revealed a significant indirect effect of resilience on negative affect through self-compassion, b = -1.03, 95% CI [-1.29, -0.80], supporting a mediation effect. The direct effect of resilience on negative affect was diminished yet remained statistically significant after accounting for the mediator, b = 0.36, 95% CI [0.01, 0.71].

**Fig. 1:**
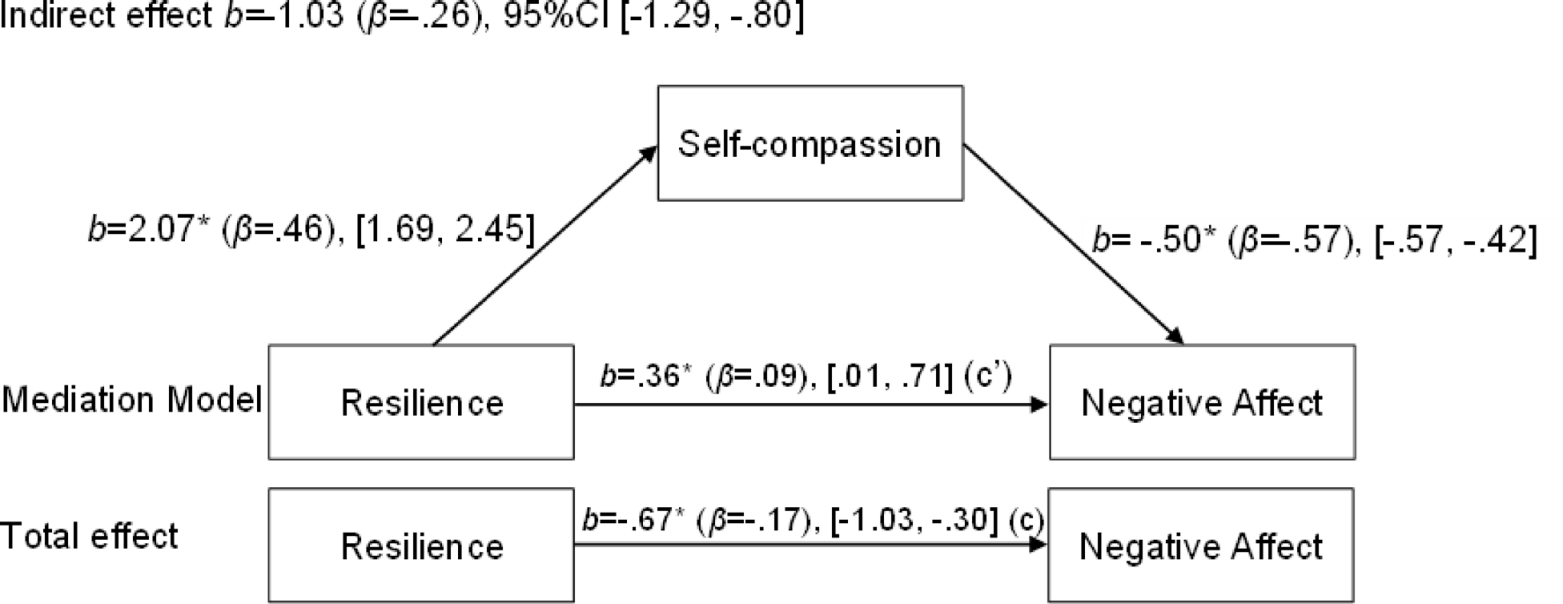
Mediation model of the relationship between resilience and negative affect, with self-compassion as the mediator.

**Fig. 2:**
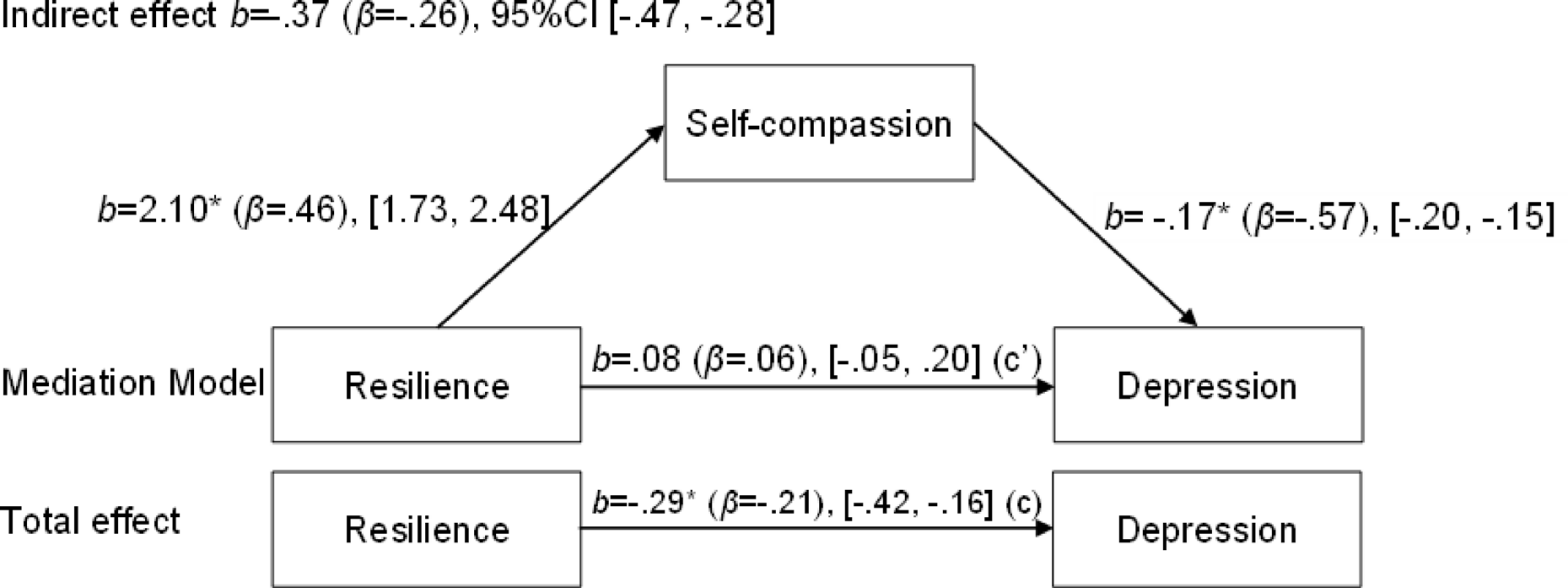
Mediation model of the relationship between resilience and depression, with self-compassion as the mediator.

**Fig. 3:**
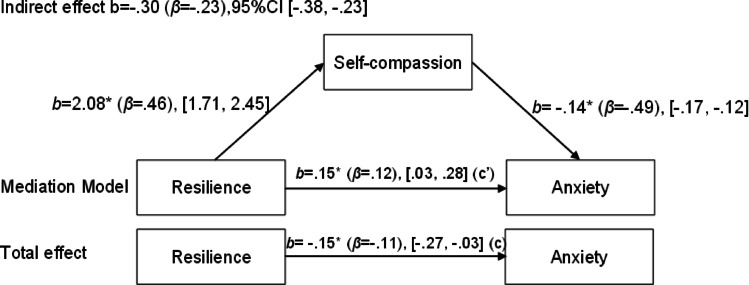
Mediation model of the relationship between resilience and anxiety, with self-compassion as the mediator.

**Fig. 4:**
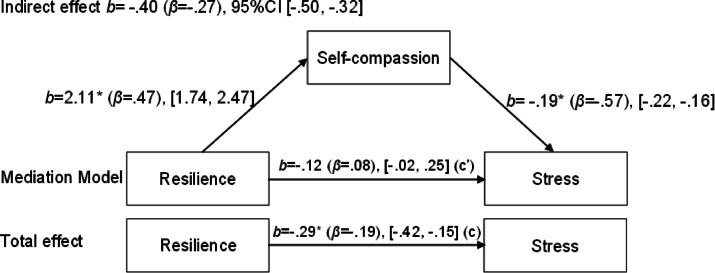
Mediation model of the relationship between resilience and stress, with self-compassion as the mediator.

Next, three additional mediation models were tested with anxiety, stress, and depression as the respective outcome variables. For depression, a significant indirect effect of resilience through self-compassion was observed (b = -0.37, 95% CI [-0.47, -0.28]), while the direct association was no longer statistically significant (b = 0.08, 95% CI [-0.05, 0.20]). For anxiety, the indirect effect through self-compassion was also significant (b = -0.30, 95% CI [-0.38, -0.23]), and the direct association remained statistically significant (b = 0.15, 95% CI [0.03, 0.28]). Finally, for stress, a significant indirect effect was found (b = -0.40, 95% CI [-0.50, -0.32]), with the direct association again being non-significant (b = 0.12, 95% CI [-0.02, 0.25]). Figures 1, 2, 3 and 4 illustrate these mediation models.

## Discussion

The objective of this study was to determine whether self-compassion mediates the link between resilience and negative affect. The primary hypothesis stated that greater resilience would be associated with lower levels of negative affect (stress, anxiety, and depression), a relationship that was empirically supported. Existing evidence suggests that resilience may partially mediate the associations between mindfulness, self-compassion, and depressive symptoms, although this does not hold for anxiety^8^. Consistent with previous literature, the results of the present investigation indicate that increased resilience is associated with fewer depressive symptoms, reduced anxiety, diminished perceived stress, and may operate as a protective factor against the development of mental health problems^33^. As resilience appears to be a reliable correlate of negative affect in the general population, it represents a useful resource for supporting psychological well-being and mental health. Resilience is significantly associated with responses to everyday stress, contributing to observable differences in emotional reactions^51^. It is associated with an enhanced capacity to manage suffering and vulnerability related to personal, familial, relational, and existential challenges and may be linked to the activation of more adaptive psychological resources^28^.

The second hypothesis proposed that self-compassion would be significantly associated with both resilience and negative affect. This hypothesis was supported and is consistent with existing research demonstrating that self-compassion predicts psychological well-being and reduces psychological distress through its positive impact on resilience^52,53^. It follows that encouraging self-compassion may help to decrease symptoms of anxiety, depression, and stress^45^. These outcomes are likely explained by enhancements in mindful awareness and the regulation of emotions, together with an increased capacity for self-compassionate behaviour, which diminishes the tendency to engage in symptom-focused rumination and both cognitive and behavioural avoidance^23,54–58^.

The third hypothesis examined whether self-compassion statistically mediated the association between resilience and negative affect. The models indicated that self-compassion was a significant statistical mediator of the relationship between resilience and all three negative affect outcomes. For depression and stress, the direct association was no longer statistically significant when accounting for self-compassion, whereas for anxiety, the direct association remained significant. These results suggest that the association between resilience and negative affect can be statistically accounted for, in part, by self-compassion. Prior research has shown that resilience mediates the relationship between self-compassion and negative affect^8,59^. Given that resilience is not solely a personality trait, but rather a multifaceted process involving behaviours, cognitions, and actions shaped by both individual characteristics and environmental factors, the present research investigated the mediating effect of self-compassion on the association between resilience and negative affect. Importantly, this research is the first to directly investigate the mediating role of self-compassion in this specific relationship.

A notable finding from the mediation analyses was the emergence of a statistical suppression effect. Although the total association of resilience with anxiety and depression was negative, the direct association was positive after accounting for the mediating role of self-compassion. This indicates that when the variance shared by self-compassion is statistically controlled for, the remaining variance in resilience is associated with increased symptom levels. A plausible interpretation is that resilience, in the absence of self-compassion, may manifest as maladaptive perseverance or emotional stoicism. Such coping styles, while potentially supporting persistence through adversity, may be insufficient to address underlying emotional distress. These results therefore suggest that self-compassion may be a critical component in the relationship between resilience and mental health outcomes; without it, the nature of the association between resilience and psychological distress appears to be reversed.

The observed statistical mediation has implications for understanding intrapersonal functioning. Self-compassion can be fostered through cognitive behavioural therapies, particularly third-wave approaches that focus on mindfulness, resilience, and self-compassion^7,8,60^. These therapeutic approaches may serve to buffer the intensity of maladaptive negative emotions. The findings suggest that self-compassion is a significant correlate and a potential statistical explanatory factor in the relationship between resilience and negative affect.

Self-compassion is recognised as a protective factor that enhances psychological well-being across the general population^21,61,62^. There is consistent evidence for positive associations between self-compassion and adaptive coping strategies, while negative associations are observed with maladaptive coping strategies^63^. Furthermore, self-compassion constitutes an active component within interventions targeting symptoms of depression and anxiety^64^.

In addition to the main effects, we observed notable gender differences in the study variables: women reported higher levels of depression, anxiety, and stress than men, whereas men reported higher self-compassion, with no significant gender differences in resilience. These patterns are broadly consistent with epidemiological data indicating higher internalising symptoms among women and suggest that men may, on average, endorse more self-compassionate attitudes^65,66^. Although sex was statistically controlled in all regression and mediation analyses, the possibility remains that the strength of the associations among resilience, self-compassion, and negative affect may differ by gender. The sample size in the present study was not sufficient to conduct well-powered multi-group mediation analyses, but future research should examine whether the indirect effects observed here are moderated by gender.

### Clinical implications

The associations observed among self-compassion, resilience, and negative affect may have clinical implications. The present findings are consistent with the view that self-compassion is associated with adaptive psychological responses to adversity. Specifically, fostering self-compassion in therapeutic settings may help individuals develop a more constructive inner voice. By encouraging self-kindness and acceptance, individuals may learn to navigate difficult emotions and experiences with greater understanding and less self-criticism. Furthermore, self-compassion is correlated with more effective emotional regulation strategies and lower reported levels of anxiety, depression and stress. Crucially, by promoting perceptions of common humanity and social connectedness, self-compassion may reduce feelings of psychological isolation and be associated with more adaptive recovery from adversity. Incorporating self-compassion training into established therapeutic approaches, particularly cognitive behavioural therapy (CBT) and mindfulness-based interventions, could be a valuable addition. Such integration would provide patients with evidence-based techniques to develop self-compassion, which may be associated with improved emotional regulation and resilience. This dual focus on skill-building and psychological flexibility may correspond to more sustainable improvements in mental health outcomes and quality of life.

### Limitations

This study has several limitations that should be considered. First, the cross-sectional design precludes any conclusions regarding causality or the directionality of the observed relationships. While our analysis was guided by a theoretical framework specifying resilience as a distal predictor, the reverse pathway, in which self-compassion fosters resilience is also theoretically sound and supported by prior research^8,67^. Therefore, the mediation effects reported in this study must be interpreted as statistical associations that are consistent with our proposed model, not as evidence of a causal chain. Disentangling the temporal precedence and dynamic interplay between these constructs require longitudinal or experimental research designs.

A second significant limitation is the use of a non-probability snowball sampling technique, which relied on university student volunteers for recruitment. This convenience-based method introduces a considerable risk of selection bias, as the sample may not be representative of the broader Greek Cypriot population. The social networks of students may over-represent individuals with similar demographic, educational, and socioeconomic characteristics, thereby limiting the generalisability of the findings beyond this specific subgroup. Consequently, the observed relationships between resilience, self-compassion, and negative affect may not hold in a more systematically recruited population-representative sample.

A third limitation stems from our reliance on self-report measures, which, despite their widespread use in psychological research, remain vulnerable to common method variance, social desirability bias, recall inaccuracies, and subjective interpretation of items^68,69^. Future studies should employ multi-method approaches, including behavioural assessments, physiological measures, or clinician-rated outcomes, to triangulate findings and provide a more robust understanding of these psychological constructs.

Fourth, the sample comprised only Greek Cypriot individuals, which may restrict the generalisability of the findings to other populations. Given that cultural norms can shape emotional expression, coping strategies, and responses to self-report, future research should examine these associations across diverse sociocultural settings to determine their broader applicability.

Furthermore, while the DASS-21, SCS, and BRS have been validated in various populations, they were primarily developed in Western societies. Their applicability to Greek Cypriot individuals, with potentially unique cultural nuances and interpretations, warrants further investigation. Future research should investigate the cultural validity and equivalence of these measures in this population.

Finally, it is important to consider the measurement of self-compassion. The Self-Compassion Scale (SCS) includes both compassionate responding (e.g., self-kindness) and uncompassionate responding (e.g., self-judgement). Although we used the SCS total score in line with the theoretical view of self-compassion as an integrated construct, some researchers have noted that the SCS negative subscales may overlap conceptually with psychopathology measures, which could inflate associations with negative affect^70^. Accordingly, the magnitude of the observed relationships should be interpreted with this potential overlap in mind. Future research should examine whether the indirect effects observed here are driven differentially by specific SCS facets (e.g., positive vs. negative components), using more granular subscale analyses and/or alternative measurement models.

## Conclusion

This study underscores the association of self-compassion with psychological well-being. Our results demonstrate a significant inverse relationship between resilience and negative affect, encompassing stress, anxiety, and depression, with self-compassion emerging as a strong correlate of both greater resilience and reduced emotional distress. Notably, we present novel evidence that self-compassion statistically accounts for a significant portion of the variance in the relationship between resilience and negative affect. These findings suggest the relevance of self-compassion-based strategies in mental health interventions and offer a promising area for further research aimed at understanding the factors associated with resilience and psychological distress.

## Data Availability

The data are available upon reasonable request from corresponding author.
